# TeaMiD: a comprehensive database of simple sequence repeat markers of tea

**DOI:** 10.1093/database/baaa013

**Published:** 2020-03-11

**Authors:** Himanshu Dubey, Hukam C Rawal, Megha Rohilla, Urvashi Lama, P Mohan Kumar, Tanoy Bandyopadhyay, Madhurjya Gogoi, Nagendra Kumar Singh, Tapan Kumar Mondal

**Affiliations:** 1 Indian Council Agricultural Research-National Institute for Plant Biotechnology, Lal Bahadur Sashtri Centre, Indian Agricultural Research Institute, Pusa, New Delhi 110012, India; 2 Darjeeling Tea Research and Development Centre, Tea Board, Ministry of Commerce, B.T.M. Sarani (Brabourne Road), Kolkata, West Bengal 700001, India; 3 Department of Biotechnology, Tocklai Experimental Station, Tea Research Association, Jorhat, Assam, India

## Abstract

Tea is a highly cross-pollinated, woody, perennial tree. High heterozygosity combined with a long gestational period makes conventional breeding a cumbersome process. Therefore, marker-assisted breeding is a better alternative approach when compared with conventional breeding. Considering the large genome size of tea (~3 Gb), information about simple sequence repeat (SSR) is scanty. Thus, we have taken advantage of the recently published tea genomes to identify large numbers of SSR markers in the tea. Besides the genomic sequences, we identified SSRs from the other publicly available sequences such as RNA-seq, GSS, ESTs and organelle genomes (chloroplasts and mitochondrial) and also searched published literature to catalog validated set of tea SSR markers. The complete exercise yielded a total of 935 547 SSRs. Out of the total, 82 SSRs were selected for validation among a diverse set of tea genotypes. Six primers (each with four to six alleles, an average of five alleles per locus) out of the total 27 polymorphic primers were used for a diversity analysis in 36 tea genotypes with mean polymorphic information content of 0.61–0.76. Finally, using all the information generated in this study, we have developed a user-friendly database (TeaMiD; http://indianteagenome.in:8080/teamid/) that hosts SSR from all the six resources including three nuclear genomes of tea and transcriptome sequences of 17 *Camellia* wild species.

Database URL: http://indianteagenome.in:8080/teamid/

## Introduction

Tea [*Camellia sinensis* (L.) O. Kuntze] is a woody plant species that belongs to the Theaceae family. Its tender leaves are used to make one of the world’s most popular non-alcoholic, caffeine-containing beverages. Tea leaves contain many secondary metabolites along with flavonoids, tannins, polyphenols, amino acids and volatile constituents that give the tea its unique aromatic and refreshing properties. The active ingredients present in tea leaves have shown antioxidant and anti-cancer activities and also help in improving cardiovascular ailments (1–4).

Based on the morphological features such as leaf size, flowers and branching, tea plants are categorized into three main taxa: China, Assam and Cambod types. Existing major economically important cultivated tea varieties are natural hybrids of these taxa (5). The quality of cultivated tea is influenced by genotype and its interaction with environmental factors such as geographical areas, altitude, soil type and weather (6). These factors affect the composition and accumulation of secondary metabolites in tea leaves that are important for the aroma and quality of prepared tea. There are approximately 600 tea varieties (7) cultivated around the world. These varieties may differ in various traits such as plant height, leaf shape and size, resistance to biotic as well as abiotic stresses and, most importantly, the accumulation of active secondary metabolites that directly affect the quality of tea. In the past, various breeding programs have been conducted to produce tea varieties with improved traits (8–12).

The breeding and development of varieties with desirable traits and enhanced characters is benefited by the availability of molecular markers linked to the trait of interest. The availability of markers allows foreground selection of donor plants, rapid screening of progenies and background selection (8,9,13–16). Various studies have reported the development and utilization of molecular markers for quality enhancements and diversity analysis of germplasm. DNA molecular markers, such as random amplified polymorphic DNA (17), amplified fragment length polymorphism (18), simple sequence repeats (SSRs) (19), inter-SSR (20), etc., have been employed to assess the genetic diversity of tea germplasm. The dominant nature of RAPD marker reported to overestimate the genetic diversity in various plants including tea (21–24). Thus, co-dominant markers such as microsatellite/SSRs offer several advantages over dominant markers as they can differentiate between heterozygous and homozygous individual and are usually highly polymorphic (25). These markers have locus specificity and higher reproducibility; in addition, SSR markers can be efficiently used in laboratories with minimum molecular biology setup in contrast to single nucleotide polymorphism (SNP) that requires specialized laboratory setups (25,26). SSR markers are highly informative and reliable for evaluating the population structures and genetic diversity of self- as well as cross-pollinating plants and also for resolving complex relationships among closely related taxa (27–31).

However, one of the prerequisites to use the marker information by the scientific community is to put them in an organized way through the creation of a database for wide access and use. SSR databases have been developed and are freely accessible for various crop species such as rice, maize and wheat (32,33). Database such as GRAMENE contains extensive information on markers for various crops (34). In tea, although various studies have reported the development of SSR markers (19,35–39), to date, no comprehensive database on SSR markers is publically available for the tea breeders. The availability of genomic resources of tea (40–42) along with robust tools and computational resources has enabled us to construct and freely provide a comprehensive database of tea SSR markers to the tea breeder’s community.

In this study, we have identified a large number of SSRs in sequenced tea nuclear and organelle genomes along with various transcriptomic resources. The nuclear SSR markers were further classified based on the motif repetition length into Group I and Group II SSRs. Annotations have been provided for the SSR-containing genes. Finally, we developed a comprehensive database named TeaMiD: Tea Microsatellite Database (http://indianteagenome.in:8080/teamid), for easy access to all this information in a user-friendly manner for the scientific community.

## Materials and Methods

### Data sources

For the present study, we have used six different data sources provided as Supplementary Table S1a and briefly described here: (i) three published tea genomes *C. sinensis* var. *assamica* (CSA) (40), *C. sinensis* var. *sinensis* (CSS) (41) and *C. assamica* (CA; TV-1) (42); (ii) assembled transcripts (TSA) form 170 RNA-seq experiments downloaded from the Sequence Read Archive (http://www.ncbi.nlm.nih.gov/Traces/sra/) as described earlier (43); (iii) tea SSR published in literatures (36,38,39,44–49); (iv) mitochondrial and chloroplast genome sequenced by our group (50) and 15 chloroplast genomes reported earlier by various groups (51–53); (v) a non-redundant set of nucleotide sequences created by assembly of ESTs, GSS and other nucleotide sequences pertaining to CSA and CSS available in NCBI nucleotide database (https://www.ncbi.nlm.nih.gov/nuccore/) until March 2019, using CAP3 software (54) with default parameters; and (vi) transcriptomic data of 17 different wild *Camellia* species that are available in the Tea Plant Information Archive database (55).

### SSR prediction and primer design

Open-source tool Krait (56) was used for the mining of SSRs from different data sources. Perfect SSRs from genomic and transcriptomic sequences were identified for five different categories, i.e. di- to hexa-nucleotide with a minimum repeat motif length of ≥18 bp. This includes di-nucleotide repeats ≥10 bp, tri-nucleotide repeats ≥6 bp, tetra-nucleotide repeats ≥5 bp and penta-nucleotide repeats ≥4 bp and hexa-nucleotide repeats of ≥3 bp. Identified SSRs were categorized into two groups: Group I, hypervariable SSRs with the motif length of ≥50 nt, and Group II, potentially variable SSRs (≥20–<50 nt motif length) (57). Primers for the predicted SSRs were designed using Primer3 software (58) implemented in the Krait tool. For primer design, 100 bp flanking sequence of identified SSRs were utilized with the following parameter: primer length of 20–25 bases with an optimum of 22 bases, polymerase chain reaction (PCR) product size range of 100–300 bp, optimum annealing temperature of 50–60°C and GC content of 40–60% with an optimum of 50%. Rest of the parameters were kept at default values in the Primer3 tool.

For the prediction of potentially polymorphic SSR among the three tea genomes, CandiSSR tool was employed with default parameters (59). We developed a linkage group for the CA genome using 6042 SNPs reported for tea in the previous study (60) and applied the methodology used for linkage group construction of the CSA genome (41). The developed linkage group of the CA genome was used as a reference for this analysis.

SSRs of organelle genomes (mitochondria and chloroplast) and transcriptomic resources of *Camellia* wild species (55) were also analyzed using Krait tool (56). For organelle genomes, a minimum length of repeat motifs were set as follows: mono-nucleotide repeats ≥8 bp, di and tri-nucleotide repeats ≥4 bp and tetra to hexa-nucleotide repeats ≥3 bp (50), while for the transcriptome sequences of *Camellia* wild species a minimum of 8 bp repeats for both di and tri-nucleotide and 3 bp repeats for tetra to hexa-nucleotide motifs were applied.

### Annotation of genes containing SSRs

To functionally annotate the nuclear genes containing SSRs, BLASTx (61) search was performed using the Swiss-Prot database with *E*-value cut-off 1e^−5^. Further, the functional domain annotations of these sequences and KEGG pathway analysis was performed using the Blast2Go tool (62).

### Validation of SSRs

For the validation of SSRs predicted in nuclear genomes of *Camellia* species, we selected 82 SSRs comprising 58 hypervariable (≥50 nt) SSR markers and 24 potentially polymorphic SSR (≥20–<50 nt) markers as predicted by the CandiSSR tool. Genomic DNA was extracted from 36 genotypes of tea following the protocol of Mondal *et al.* (63). Primers used for validation are listed in Supplementary Table S1b. PCR reactions were performed as described earlier (64). The molecular weight marker (100 bp ladder) was used to identify the molecular weight of the amplified products. The gel was stained with ethidium bromide and viewed under the Gel Doc system (Gel Doc XR^+^ system, BioRad, USA). The number of alleles and the polymorphic information content (PIC) was estimated for each SSR using Darwin 6 software (65).

### Database design

The organization of this database is based on a ‘three-tier’ system. They are client tier, middle tier and database. The PHP programming language is used here for connecting the client tier with the database. It was designed using phpMyAdmin (www.phpmyadmin.net). The client tier was created using HTML, CSS and Bootstrap. The information then stored in a web-enabled database entitled ‘Tea Microsatellite Database (TeaMiD)’ is hosted at National Institute for Plant Biotechnology, New Delhi, India. SSRs identified from all the resources have been compiled in the form of a database for easy access and retrieval.

## Results

### SSR mining in CSA, CSS and CA nuclear genomes

**Figure 1:**
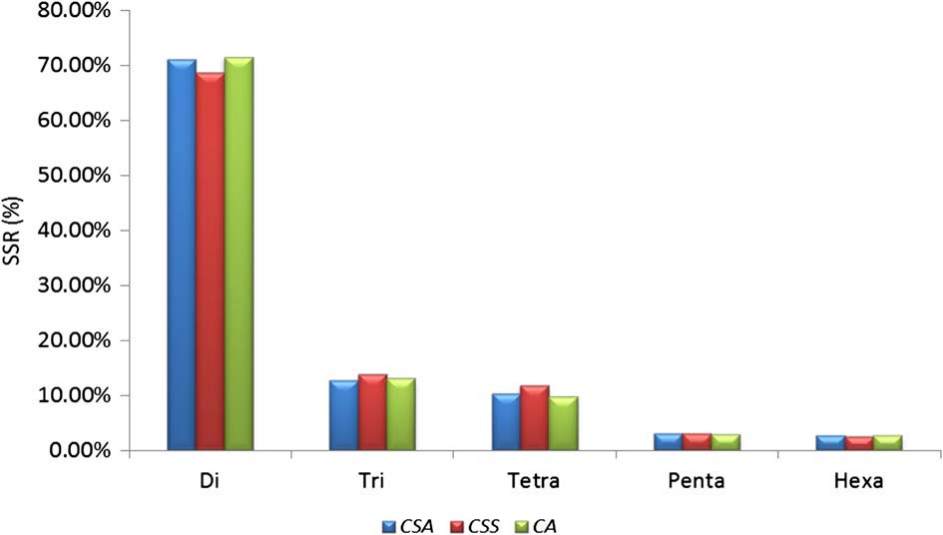
Comparative analysis of SSR in nuclear genomes of *Camellia* species. Frequency of di- to hexa-nucleotide repeat motifs detected in the three *Camellia* species, namely CSA, CSS and CA.

A total of 239 011 SSRs were identified in CSS genome (41) followed by 193 911 SSRs in CA (42) and 166 993 SSRs in CSA (40). Mono-nucleotide repeats, complex SSRs and SSR loci with length <18 bp were not included in this study. Di-nucleotide repeats were identified as the most abundant SSRs with 71.13% (118 777), 68.61% (163 982) and 71.52% (138 689) in CSA, CSS and CA genomes, respectively. Tri-nucleotide motifs comprised the second largest proportion (12.79%, 13.90% and 13.09% in CSA, CSS and CA, respectively), followed by tetra-nucleotide SSRs (10.24%, 11.89% and 9.71% in CSA, CSS and CA, respectively). We observed smaller frequencies of penta-nucleotide (3.10%, 3.05% and 2.95% in CSA, CSS and CA, respectively) and hexa-nucleotide (2.75%, 2.55% and 2.72% in CSA, CSS and CA, respectively) SSRs in the analyzed genomes. (Table 1; Figure 1; Supplementary Table S2a).

**Table 1:** Characteristics of identified SSRs in the three *Camellia* genomes

Species	CSA (40)		CSS (41)		CA (TV-1)	
	Number	%	Number	%	Number	%
Di	118 777	71.13%	163 982	68.61%	138 689	71.52%
Tri	21 352	12.79%	33 223	13.90%	25 392	13.09%
Tetra	17 096	10.24%	28 426	11.89%	18 829	9.71%
Penta	5183	3.10%	7289	3.05%	5720	2.95%
Hexa	4585	2.75%	6091	2.55%	5281	2.72%
Total	166 993	100%	239 011	100%	193 911	100%
Group I (hypervariable SSRs ≥ 50 nt)	2288	1.37%	4574	1.91%	3445	1.77%
Group II (potentially variable SSRs, ≥20–< 50 nt)	164 705	98.62%	234 438	98.08%	190 466	98.22%

**Figure 2:**
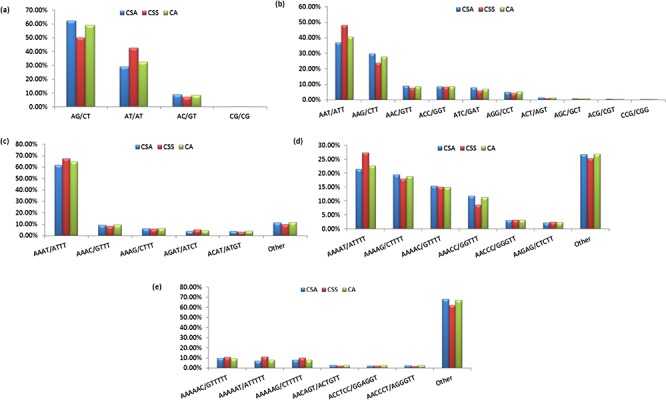
Frequency of identified motifs and their complementary sequences in the nuclear genomes of *Camellia* species. Figure 2(**a–e**) represent the frequency of di- to hexa-nucleotide repeat motifs and their complementary sequences in CSA, CSS and CA, respectively.

**Figure 3:**
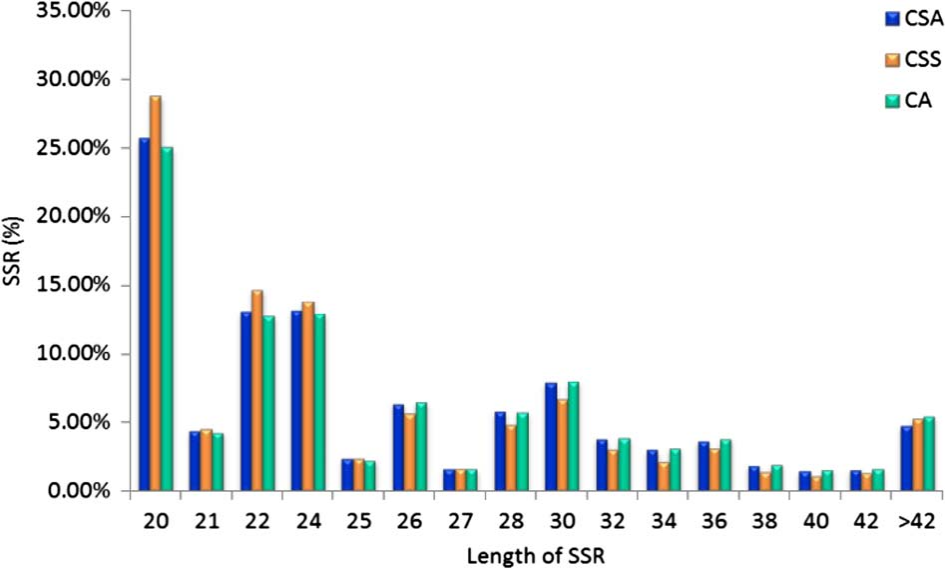
Length distribution of SSRs identified in the nuclear genomes of *Camellia* species, CSA, CSS and CA.

Specifically, among the di-nucleotide repeat motif, AG/CT (50.09% in CSS, 58.92% in CA and 62.22% in CSA) and AT/TA (42.68% in CSS, 32.55% in CA and 28.91% in CSA) were identified as a major/dominant motif followed by AC/GT (8.86% in CSA, 7.22% in CSS and 8.52% in CA) and CG/CG motif was identified with the least number (0.01% each in CSA, CSS, CA) in all three genomes (Figure 2a; Supplementary Table S2b). From the set of tri-nucleotide repeat motif, AAT/ATT (36.86% in CSA, 48.07% in CSS and 40.60% in CA) and AAG/CTT (29.75% in CSA, 23.75% in CSS and 27.69% in CA) were present with the highest proportion in all the three genomes and CCG/CGG motif was identified with the lowest proportion with 0.28% in CSA genome and the same pattern was followed in the remaining two *Camellia* species (0.24% in CSS and 0.26% in CA) (Figure 2b; Supplementary Table S2b). The most abundant SSRs among the tetra-nucleotide motifs were AAAT/TTTA (64.42% in CSA, 67.43% in CSS and 64.58% in CA) in all genomes (Figure 2c; Supplementary Table S2b). Among the penta-nucleotide and hexa-nucleotide motifs of SSR, AAAAT/TTTTA (21.47% in CSA, 27.37% in CSS and 22.67% in CA) and AAAAAC/GTTTTT (9.61% in CSA, 10.62% in CSS and 9.2% in CA) were identified (Figure 2d and e; Supplementary Table S2b). In addition, the most abundant SSR length was 20 bp accounting for 25.64%, 28.73% and 24.98% of the total SSRs in CSA, CSS and CA genomes (Figure 3). The second most abundant SSR length was found to be 24 bp in CSA and CA genomes (13.10% and 12.91%, respectively) followed by 22 bp (13.03% and 12.74%, respectively) while in CSS genome, SSRs containing a length of 22 bp were present with the high proportion (14.60%) in comparison with 24 bp (13.81%) SSR length (Figure 3).

### *In silico* prediction of potentially polymorphic nuclear genomic SSRs in tea

We developed linkage groups for the CA genome, as described in Materials and Methods, to identify linkage group-wise SSR markers in the CA genome that may also show polymorphism among the three tea genomes (CA, CSA and CSS). We utilized the CandiSSR tool (59) for this purpose. This tool takes two or more sequence files, identifies SSRs in the designated reference genome and/or transcriptomic sequence file, designs primers for the identified SSRs and then compares the primer binding sites in the other provided input sequence files to assess the cross-transferability of the designed markers. In this analysis, we used linkage groups developed for the CA genome as a reference to predict potentially polymorphic SSR and their transferability in the other two genomes. A total of 33 991 candidate polymorphic SSRs were identified and primers were designed for 90.27% (30685) of SSRs (Supplementary Table S3).

### Nuclear genomic SSR overlapping with genes of CSS and CSA genome

To identify the SSR overlapping with the *Camellia* genes, we used two publically available genomes with associated gene models. The overlap between SSR and gene loci was identified using intersectBed function available in the BEDtools (66) with default parameters. Out of the total predicted SSRs in CSS and CSA genomes, 33 054 and 14 635 SSRs were identified to overlap with 16 053 and 9341 genes in the respective genomes. The annotation of SSR-containing genes was performed to identify the pathways associated with these genes. Significant hits for 13 798 (85.95%) and 7678 (82.19%) SSR-containing genes were obtained against the Swiss-Prot database from CSS and CSA genomes, respectively. These genes were found to participate in a total of 143 and 125 pathways in CSS and CSA genomes, respectively (Supplementary Table S4a and b). A total of 5051 (31.46%) genes out of the total SSR-containing genes were annotated with 752 unique enzyme accession in the CSS genome. Among the genes annotated as enzymes, the highest numbers of annotations were obtained for EC:3.6.1.15—phosphatase (875) and EC:3.6.1.3—adenyl pyrophosphatase (633) participating in thiamine metabolism and purine metabolism, respectively (Supplementary Table S4c). In CSA, a total of 1491 (15.96%) genes were annotated with 408 unique enzyme accession codes (Supplementary Table S4d).

Some of the SSR-containing genes were found to participate in the pathways that directly affect the tea quality such as caffeine metabolism, flavonoid biosynthesis, isoflavonoid biosynthesis, flavone and flavonol biosynthesis, anthocyanin biosynthesis and other active secondary metabolites (Supplementary Table S5). Compared with CSA, higher numbers of SSR-containing genes from these pathways were annotated as enzymes in the CSS genome. The reason for the difference in the number of annotated enzymes were (i) many of these genes do not have SSR in the CSA genome and (ii) some of the genes were present in the higher copy numbers in CSS as compared with CSA such as enzyme EC:1.11.1.7—lactoperoxidase has 81 copies in CSS while 19 were present in CSA (Supplementary Table S5).

### SSR mining from transcriptome data

**Figure 4:**
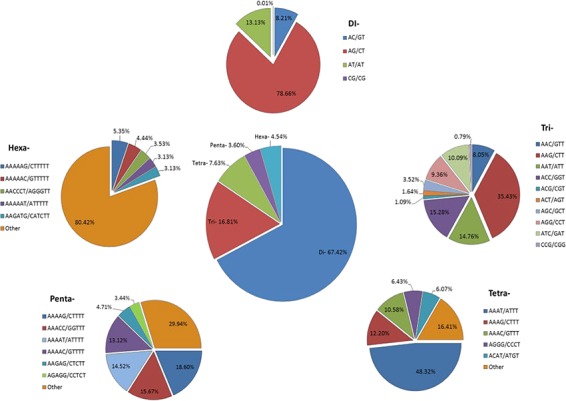
Identification of SSRs in the Transcriptome Shotgun Assembly of SRA data. Frequency of di- to hexa-nucleotide repeats in the central, large pie chart and small peripheral pie charts represent the motifs and their complementary sequences of identified di- to hexa-nucleotide repeats, respectively.

We identified 21 809 microsatellites (Supplementary Table S6a) from 123 145 TSA (with a length of ≥200 nt). These transcript sequences were obtained from the assembly of 170 RNA-seq data downloaded from the NCBI-SRA database. These RNA-seq data represent distinct tissues of the tea plant (seeds, root, stem, axillary bud, a bud and a leaf, a bud and two leaves, apical bud and two leaves, second leaf, fourth leaf, sixth leaf and flowers) from 18 different bioprojects, containing around 7157 million high-quality reads. More details about data processing and transcriptome assembly can be found in Varshney *et al.* (43). We excluded mono-nucleotide repeats and complex SSR from this study. Among the SSR-containing contigs, 14 102 (64.66%) possessed single SSR loci, while 3335 contigs (15.29%) had 2–4 SSR loci followed by 21, 8, 6 and 1 contig that had 5, 6, 7 and l0 loci. Among the different motif sizes, di-nucleotide repeats (67.42%) were dominant over the other types of repeats, followed by tri- (16.81%), tetra- (7.63%), hexa- (4.54%) and penta-nucleotide repeats (3.60%) (Figure 4; Supplementary Table S6b). The number of reiterations of a given repeat unit varied from 5 to 76, and SSRs with 10 reiterations were the most abundant (19.36%) among all the SSRs followed by 11 (13.29%) and 5 (11.21%).

**Figure 5:**
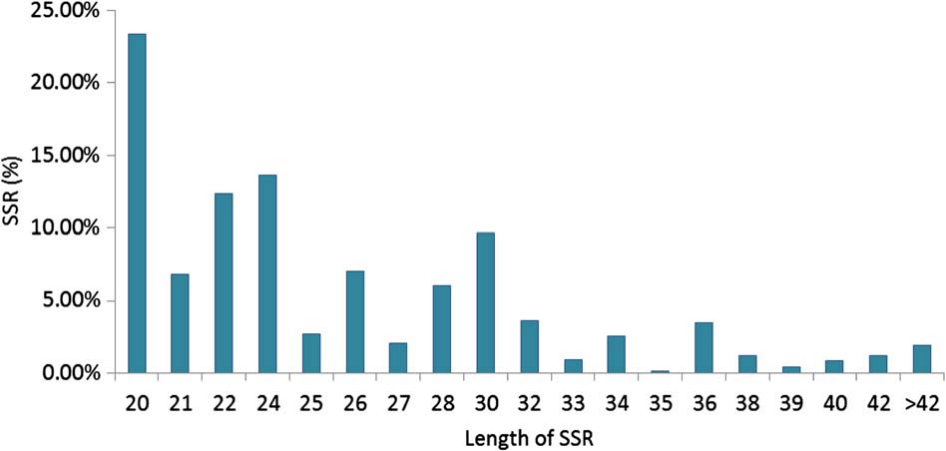
Length distribution of SSRs identified in the Transcriptome Shotgun Assembly of *Camellia*.

Among the dinucleotide repeats, AG/CT had the highest occurrence (78.66%), followed by AT/AT (13.13%) and AC/GT (8.21%) (Figure 4). Among the tri-nucleotide repeats, AAG/CTT motifs were presented with the highest proportion (35.43%), followed by ACC/GGT (15.28%) and AAT/ATT (14.76%). The most common tetra-nucleotide repeats were AAAT/ATTT (48.32%), AAAG/CTTT (12.20%) and AAAC/GTTT (10.58%). AT-rich repeat patterns were the most abundant among penta- and hexa-nucleotides, such as AAAAG/CTTTT, AAACC/GGTTT and AAAAT/ATTTT for penta-nucleotides and AAAAAG/CTTTTT, AAAAAC/GTTTTT and AACCCT/AGGGTT for hexa-nucleotides (Figure 4; Supplementary Table S6c). In addition, the most abundant SSR length was 20 bp (5087, 23.32%) followed by 24 bp (2969, 13.61%) and 22 bp (2688, 12.32%) of the total SSRs in TSA contigs (Figure 5).

A total of 289 666 SSRs (di- to hexa-nucleotides) were mined from the transcript sequences of 17 wild *Camellia* species with maximum (23 489) in *C. reticulata* and minimum (3878) in *C. leptophylla* (Supplementary Table S7). Similar nucleotide repeat frequencies were observed among these *Camellia* wild species with either tri- or tetra- as the most frequent SSR motif type, except *C. sasanqua* that has di-nucleotide SSR motifs as the most frequent SSR motif.

### Identification of hypervariable SSRs

SSRs were classified into two groups, based on the total length of the SSR motif as described by Singh *et al.* (57). Group I or hypervariable SSRs are defined with a motif length of ≥50 bp, whereas Group II or potentially variable SSRs contains a motif of ≥20 bp - <50 bp. In the CSS (41) genome, a total of 4574 (1.91%) Group I (hypervariable) SSRs were identified and primers were successfully designed for 2210 hypervariable SSRs (Supplementary Table S8a). While in the CA (42) and CSA (40) a total of 3445 (1.77%) and 2288 (1.37%) Group I SSRs were identified respectively (Supplementary Table S8b and S8c). The remaining SSRs were assigned to Group II SSRs (contained ≥20 but <50 nucleotides) in all the three genomes (Table 1). In the TSA contig, out of the total 21 809 microsatellites, only 151 SSRs were identified as hypervariable SSRs and primer designing was successful only for 120 of these microsatellites repeats (Supplementary Table S8d).

### SSR prediction in mitochondrial and chloroplast genomes

**Figure 6:**
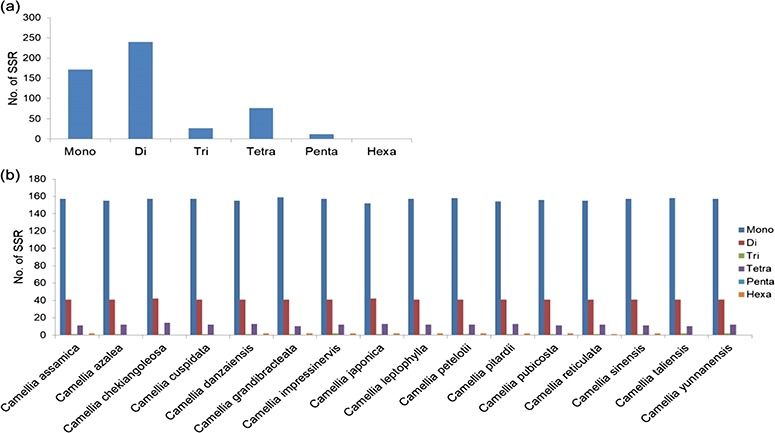
Identification of SSRs in organelle genomes of *Camellia*: (**a**) frequency of SSRs identified in mitochondria and (**b**) chloroplast genomes of 16 *Camellia* species.

A total of 529 SSRs were identified in the mitochondrial genome of CA and the overall frequency of di-nucleotide repeats was higher as compared with the other microsatellites (Figure 6a). Among the mono-nucleotide SSRs, ‘T’ motif (45.34%) was the most frequent, while in di-nucleotide SSRs, ‘AG’ (22.5%) was more prevalent. Out of the total identified SSRs, successful primers were designed for 522 microsatellites (Supplementary Table S9a and b).

Chloroplast genomes for 15 different *Camellia* species were downloaded from the public domain and 1 chloroplast genome decoded by our group (50) was also used for SSR predictions. The total numbers of SSR identified in *Camellia* chloroplast genomes ranged from 209 to 214 (Supplementary Table S9c, d and e). Mono-nucleotide SSRs were the most abundant SSRs among all analyzed species (Figure 6b; Supplementary Table S9c and d) and are dominated by the ‘T’ motif while in di-nucleotide AT followed by TA were the most frequent SSR motifs. Only few SSRs (1–3 SSRs per genome) were found in tri, tetra and hexa categories, whereas no SSR in penta-nucleotide category were identified in any of the analyzed chloroplast genomes (Supplementary Table S9c).

### Compilation of experimentally validated set of SSRs from the published literature

**Figure 7:**
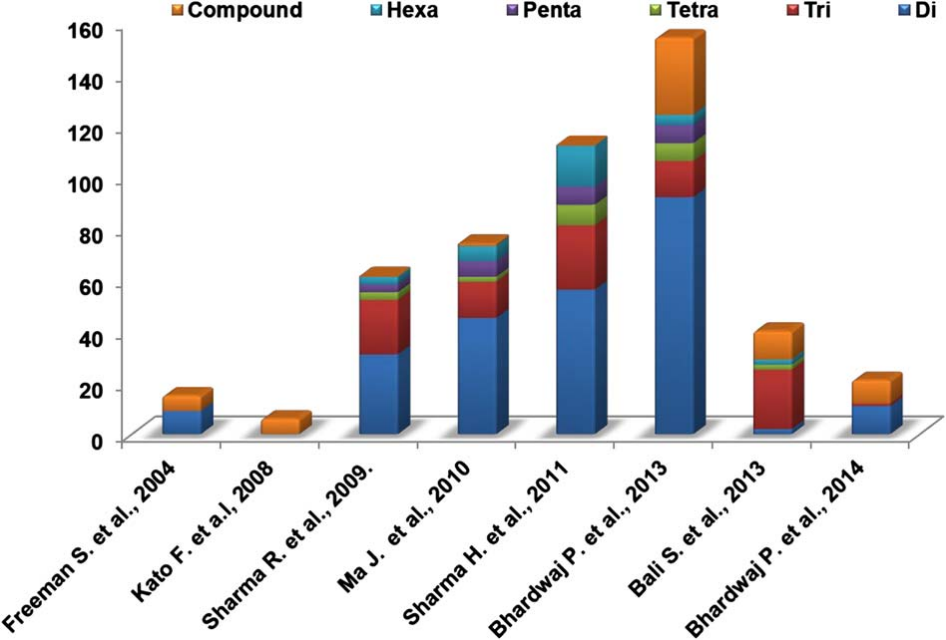
Distribution of different experimentally validated SSR in tea.

We performed the literature survey to mine the SSR markers already reported for *Camellia* species. These SSR markers have been identified from various sources like unigene-derived SSRs (38,48), ESTs (46) and genomic SSRs (36,39,44–45,49). The different types of SSR markers identified and reported in various studies are depicted in Figure 7. These markers have been utilized for population diversity analyses and genotyping of various *Camellia* species. Validated sets of SSR markers from these studies provide a valuable source for tea breeders and hence we included the information of these markers in our database (Supplementary Table S10).

### SSR from combined ESTs, GSS and other nucleotides

From the CAP3 assembled non-redundant nucleotide data (total, 46 579 contigs) of different *Camellia* species, a total of 18 031 SSRs were identified with the highest frequency for tri-nucleotide repeats (37.89%) followed by di- (29.10%) and tetra-nucleotide repeats (25.82%). The motifs ‘TCTC’ and ‘AAAAT’ were found with the highest occurrences in tetra- and penta-nucleotide SSR sets, respectively. Further, the primers were designed successfully for 18 031 SSRs (Supplementary Table S11).

**Figure 8:**
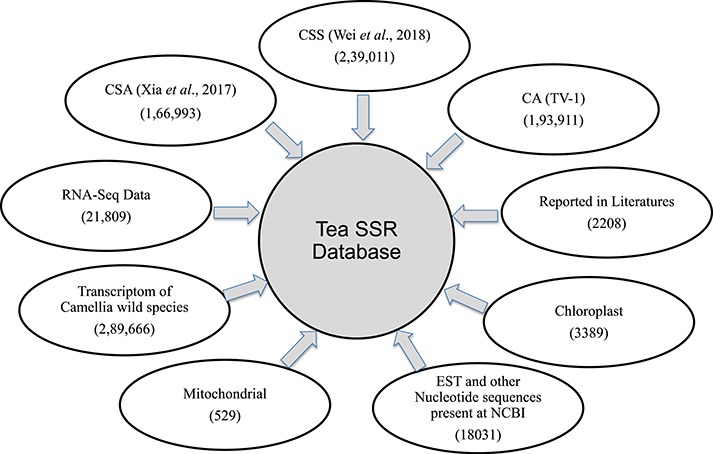
Summary of SSR database. Numbers of SSR identified and their sources used for database development.

### PCR validation of SSRs

We selected 82 SSRs (Supplementary Table S1b) comprising 58 hypervariable (≥50 nt) SSR markers and 24 potentially polymorphic SSRs (≥20 nt) as predicted by the CandiSSR tool. Genomic DNA was extracted from 36 tea genotypes (Supplementary Table S12: Supplementary Figure S1). Initially, nine tea genotypes were selected to screen the primers that yielded 27 polymorphic primers. Further, to test the degree of polymorphism, six primers (Supplementary Table S13; Figure S2) were selected for the diversity analysis in 36 tea genotypes. A total of 30 alleles were detected by these six SSR markers. The number of alleles per locus generated by each marker varied from four to six alleles, with an average of five alleles per locus. The highest number of alleles detected was at the loci TKM 1383 and TKM 1384 combination. The PIC value for these six markers varied from 0.61 to 0.76; we found the highest PIC value for TKM 1361 and TKM 1362. These SSR markers were highly informative and polymorphic as evident from their PIC value. The PIC value is a measure of polymorphism among different accessions for a marker locus. Markers with PIC value greater than 0.5 is considered as highly informative (67); hence, these six markers were used for the diversity study among the 36 different tea genotypes.

**Figure 9:**
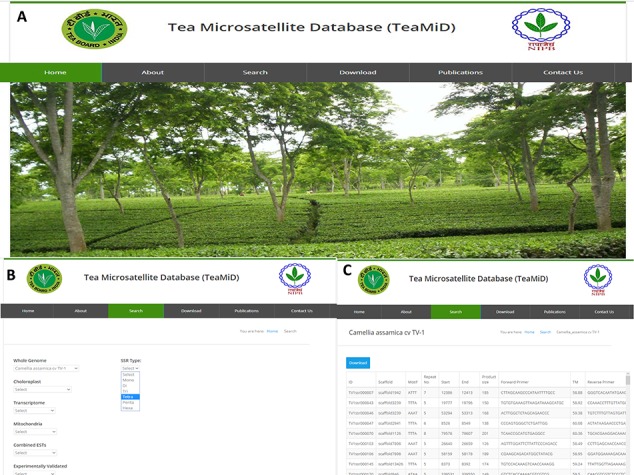
Database of tea SSR (TeaMiD) (**a**) front page of the database, (**b**) ‘Search’ page of the database indicating various option, (**c**) example of CA SSR page under the ‘whole genome’ search option.

### Database of SSRs

We have developed a database (TeaMiD; http://indianteagenome.in:8080/teamid/) that hosts the SSR from all the resources including SSRs from the nuclear genomic and also transcriptomic sequences of 17 *Camellia* wild species (Figure 8). From these resources, we have identified a total of 935 547 SSRs and made them available for the research community in the form of a user-friendly database entitled TeaMiD. Home page of the database contains six navigation options these are the `Home, About, Search, Download, publications and Contact Us (Figure 9a). `About' section provides a brief detail about the database. SSR information generated and collected from the different resources in this study can be viewed and downloaded from the `Search' menu. The `Search, page is further categorized into six options these are the `Whole Genome', `Chloroplast', `Transcriptome', `Mitochondria', `Combined ESTs' and `Experimentally Validated'. Under the `Whole Genome', `Chloroplast', `Transcriptome', `Mitochondria' and `Combined ESTs options user can select the available *Camellia* species for viewing and downloading the details on the different kinds of SSRs (di to hexa-nucleotide), their location on the genome and the details of primer sequences generated for the SSR.

**Figure 10:**
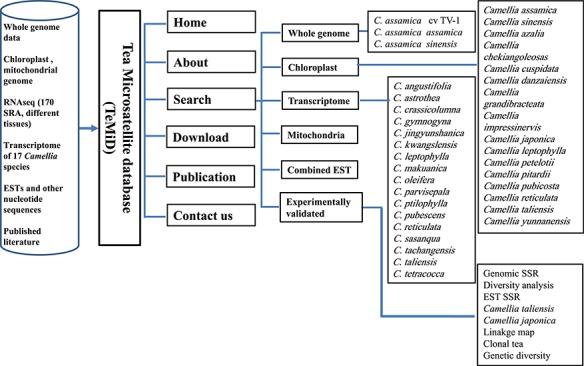
The structure of the SSR database (TeaMiD).

## Discussion

Tea leaves are the main constituent of the world’s most popular caffeine-containing beverage and is predominantly grown in Asian countries like China, India and Japan with a relatively less contribution from African and South American countries. All tea varieties grown worldwide originated either from China or India (68–71). Tea tree is an outcrossing species and it has a long breeding cycle. Developing a systematic mapping population through homozygous lines, is a difficult task in the tea. Hence, pseudo-test cross population is predominantly utilized for the quantitative trait locus (QTL) discovery and analysis (12,72). This limits the discovery of QTLs associated with important traits that directly affect the quality and thereby economics of tea. The main breeding approaches practiced for improvement of tea include the selection of promising individuals obtained from natural or controlled pollination and clonal propagation of elite individuals (73). Drinking quality of tea is the most important trait selected for tea improvement programs though yield is simultaneously considered to be important for profitability. Due to breeding constraints, only country-specific elite varieties are selected as breeding material that narrows the genetic diversity of available breeding populations (74).

Various studies have reported the development and use of SSR markers for the diversity analysis but their application in marker-assisted tea improvement is very limited. Taniguchi *et al.* (74) have analyzed the genetic diversity of tea using a subset of 788 accessions from the total 7800 worldwide accessions present at the NARO Institute of Vegetable and Tea Science, Japan, using 23 SSR markers. EST-SSR markers have also been developed and utilized for genetic diversity and population structure analysis using 450 tea accessions from China (37). A recent study has reported a large number of SSR markers using the published genomes of ‘Shuchazao’ variety tea (75,76). They have used 96 highly polymorphic SSR markers to evaluate the genetic diversity of 47 tea cultivars. Liu *et al.* (76) also reported the development of 36 highly polymorphic SSR markers from tea and evaluated their effectiveness in the population diversity analysis. Several other studies also reported the use of SSR markers for the evaluation of tea germplasm (36,38,45,46,77).

Moreover, attempts have been made to construct a linkage map of *Camellia* sps. by utilizing the information available from SSR markers and use these markers for QTL analysis. Tan *et al.* (78) generated 2439 SSR markers from unigene sequences obtained from floral transcriptome and constructed a linkage map based on 237 SSR markers covering 1156.9 cM of *Camellia* genome. Similarly, Ma *et al.* (8) have reported *Camellia* linkage map based on pseudo-testcross population utilizing 406 SSR markers derived from unigene sequences and identified nine stable QTLs associated with catechins contents spread over four linkage groups. SSR markers require a mapping population that is a serious limitation for outbreeding plants like tea. In these situations, alternative approaches such as a linkage disequilibrium-based association analysis could be advantageous as it can benefit from the available natural variations. However, this approach requires highly abundant markers such as SNP. Presently, SNP information on tea is very limited (60). In this situation, the SSR marker will be of great importance. In a recent study, SSR and SNP markers were utilized to identify QTLs associated with the accumulation of caffeine and theobromine contents in the tea plant (12). With the recent draft genome sequences of tea (40,41,42), along with the other large number of different types of sequences (36,38,39,44–53,55), we developed and hosted a comprehensive database of tea SSR on the public domain for tea breeder/researcher community. Here, we report an exhaustive database of *Camellia* SSRs extracted from nuclear and organelle genomes (chloroplast and mitochondrial) as well as the information available in the literature. In this database, users can easily get the SSRs from different sources for specific use.

Our results demonstrate that the overall frequency of the di-nucleotide repeats for the nuclear genomic SSRs was higher in comparison with the other SSR classes in all the genomes. This was corroborated with earlier reports for CSA and CSS genomes (40,41). However, the reported numbers of different classes of SSRs varied among the CSA and CSS, which could be attributed to the different sets of parameters used for the motif detection in the respective genomes (40,41). To alleviate this bias in prediction, we re-analyzed the data of the two earlier published genomes (40,41) along with CA, using the same set of parameters (refer to Materials and Methods) with the Krait tool (56). The result of this re-analysis confirmed the dominance of di-nucleotide repeats in all the genomes (71.13%, 71.52% and 68.61%, in CSA, CA and CSS, respectively) (Table 1, Figure 1; Supplementary Table S2a). Motif AG/CT within the di-nucleotide repeat was the most frequent among the others (50.09% in CSS, 58.92% in CA and 62.22% in CSA) (Figure 2a; Supplementary Table S2b). Moreover, we categorized the nuclear genomic SSRs into hypervariable (≥50 nt) and potentially variable SSRs based on the SSR length (≥20 – <50 nt). Hypervariable SSR markers have been reported to provide a higher level of polymorphism as compared with random SSR markers and can be easily scored using agarose gel electrophoresis (57,79). We also identified the gene models of CSS and CSA genome overlapping with the predicted nuclear genomic SSRs. A total of 13.82% (33 054) and 8.76% (14 635) SSRs from CSS and CSA were found to overlap with 16 053 and 9341 genes models in their respective genomes. Functional annotation of these genes revealed the participation of some of the genes in the biochemical pathway that may affect the drinking quality of prepared tea (Supplementary Table S5).

We also searched for potentially polymorphic SSRs *in silico* using the CandiSSR among the selected *Camellia* genomes (CA, CSA and CSS), which yielded a total of 30685 potentially polymorphic SSRs (Supplementary Table S3). These potentially polymorphic SSRs could be the best candidates to look for polymorphism among the *Camellia* sps. Identification of SSR in the TSA contigs from 170 *Camellia* SRA data yielded a total number of 21 809 microsatellites (Supplementary Table S6a) after removing mono-nucleotide and complex SSRs. In consistence with the previous (78,80) studies, we also observed a higher frequency of di-nucleotide repeats (67.42%) followed by tri-nucleotide repeats (16.81%) in this data set (Figure 4; Supplementary Table S5b).

In this study, we observed highly similar trends for the identified SSRs among the CSA and CA as compared with CSS, whether it is the frequency of nucleotide repeats, motif types or length distribution of SSRs in the nuclear genome (Figures 1–3), suggesting close phylogeny between CSA and CA, in comparison with CSS. Even the highly similar trends for motif type distribution among all 16 chloroplast genomes (Figure 6b) signify the conserved nature of chloroplast sequences.

In summary, we created a comprehensive database of tea SSRs from six different types of sources. Although the predominant number of SSRs are from the genomic resources of three *Camellia* species (CSA, CSS and CA), inclusion of SSRs from transcriptome sequences of 17 wild *Camellia* species, *Camellia* organelle genomes and, most importantly, SSRs from published literature provides the database a wider coverage. To our knowledge, this is the first large-scale SSR database of tea. We have also made an attempt to anchor the SSRs in the linkage map. Interestingly, we found several SSRs that were present in the transcripts involved in aroma formation pathways. These transcripts would be ideal to utilize as candidate genes in tea breeding programs. Polymorphism present in these transcripts could be further evaluated and tested for association with the phenotypic variance of the trait. This approach has been successfully employed in the improvement of various crops such as rice (81), wheat (82), potato (83), etc. The knowledge generated in this study will be helpful to tea breeders, as well as to biomedical researchers studying woody perennial plant species.

## Authors’ contributions

T.K.M. conceived and designed the work. H.D. performed prediction of SSR in various data sources and annotations of SSR overlapping genes. H.C.R. performed SSR identification in mitochondria and chloroplast genomes. M.R. and U.L. validated the SSR markers. T.B. supplied the leaf material. P.M.K., N.K.S. and M.G. provided valuable suggestions during the project execution. N.K.S. guided the work. H.D., T.K.M. and H.C.R. wrote the manuscript.

## Supplementary Material

Supp_baaa013
