# Supplementary material for: TeaMiD: a comprehensive database of simple sequence repeat markers of tea
Source: Database (Oxford). 2020 Mar 11;2020:baaa013. doi: 10.1093/database/baaa013 (PMC7065459; doi:10.1093/database/baaa013)
Supplement: Supp_baaa013 [file supp_baaa013.zip › Supplimentray Fig 1.docx]

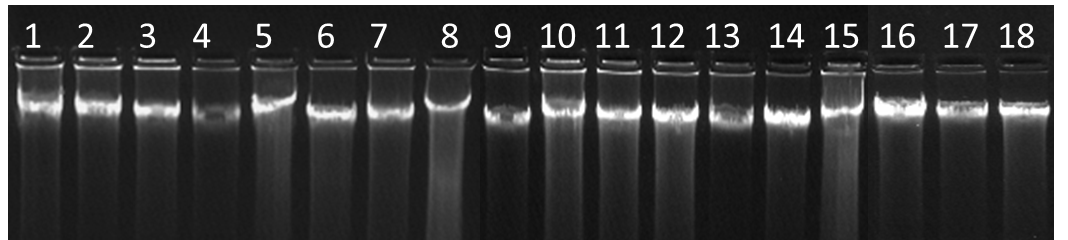


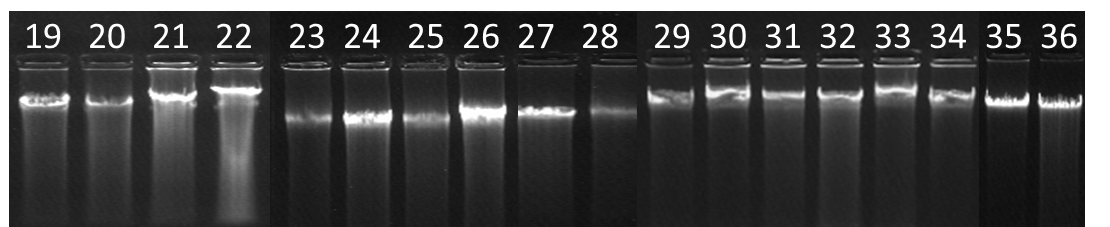


Supplementary figure S1: Genomic DNA isolated from tea 36 genotypes.

1. (CP1) China Assam; 2. (19.55.49) Assam hybrid; 3. (19.60.10) Assam hybrid; 4. (19.58.17) Assam hybrid; 5. (19.56.27) Assam hybrid; 6. (S.A.) Assam China; 7. (19.56.42) Assam hybrid; 8. (19.57.36) Assam hybrid; 9. (19.55.46) Assam hybrid; 10. (651.63) Assam hybrid;11. (651.25) Assam hybrid; 12. (P.133) Assam; 13. (LV.18)Assam; 14. (19.61.25)Assam hybrid; 15. (651.32) Assam hybrid; 16. (651.36) Assam hybrid; 17. (19.60.39) Assam hybrid; 18. (651.57) Assam hybrid; 19. (19.60.45) Assam hybrid; 20. (19.61.4)Assam hybrid; 21. (19.61.33) Assam hybrid; 22. (651.71)Assam hybrid; 23. (19.59.20)Assam hybrid; 24. (T.3E.3) Assam hybrid; 25. (19.56.21) Assam hybrid; 26. (*Camellia rosiflora*) Camellia species; 27. (SS.28)AssamChina hybrid; 28. (Tingamira) Assam hybrid; 29. (Dhonjan) Assam hybrid; 30. (128.32.8)China type; 31. (128.26.2)China type; 32. (270.3.11) Cambod hybrid; 33. (Gourisankar) Assam hybrid; 34. (*Camellia sasanqua*) Wild Camellia species.
