# Supplementary material for: TeaMiD: a comprehensive database of simple sequence repeat markers of tea
Source: Database (Oxford). 2020 Mar 11;2020:baaa013. doi: 10.1093/database/baaa013 (PMC7065459; doi:10.1093/database/baaa013)
Supplement: Supp_baaa013 [file supp_baaa013.zip › Supplimentray Fig 2.docx]

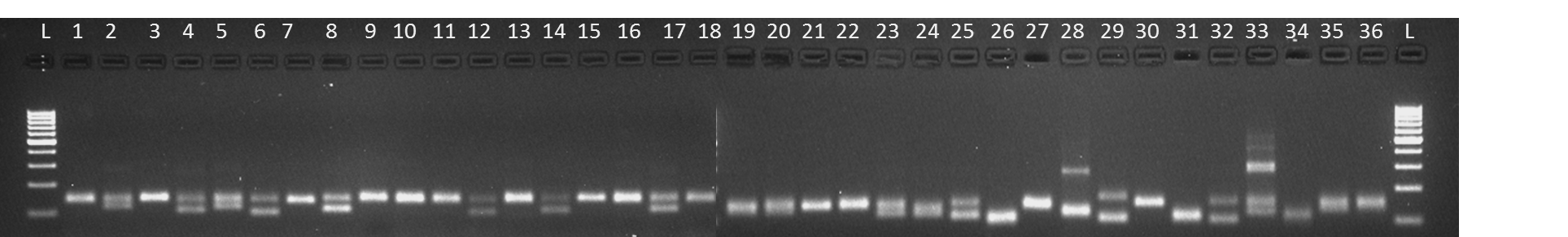

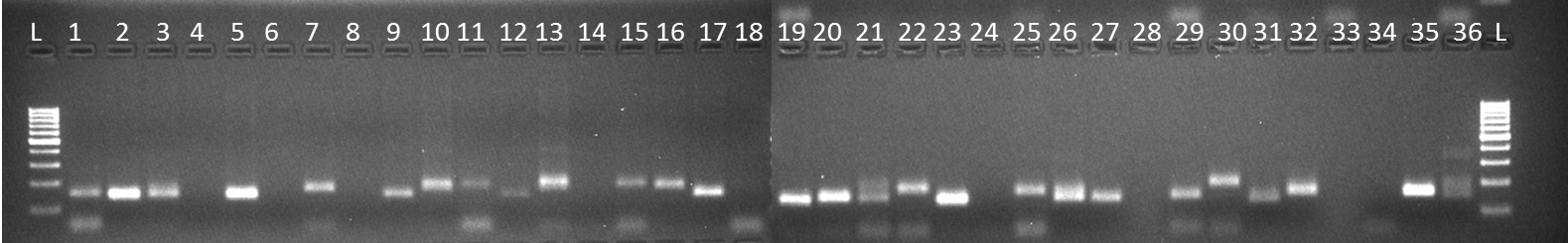

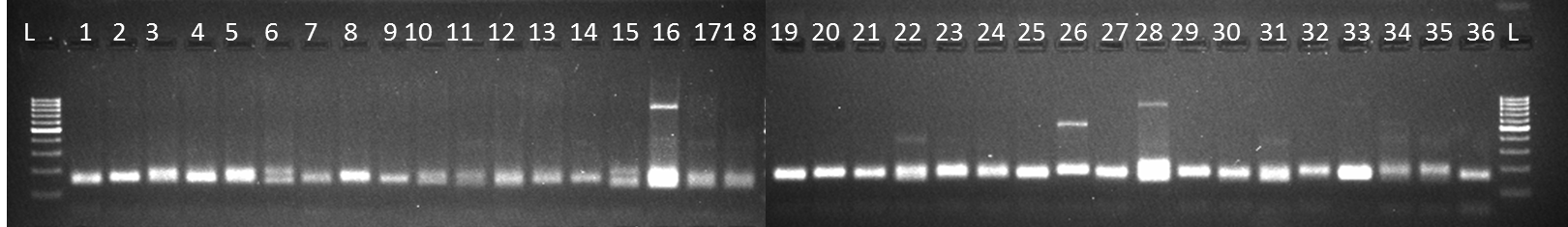


PCR amplification of 36 genotypes with primer number- TKM 1383-1384

PCR amplification of 36 genotypes with primer number- TKM 1421-1422

PCR amplification of 36 genotypes with primer number- TKM 1439-1440


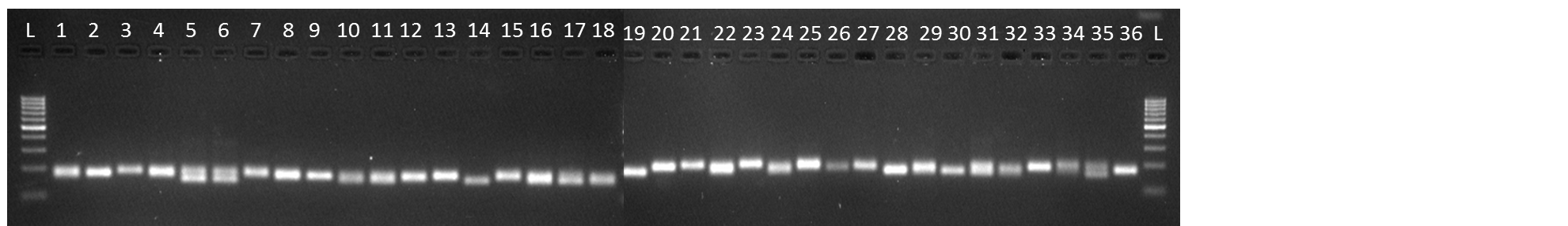

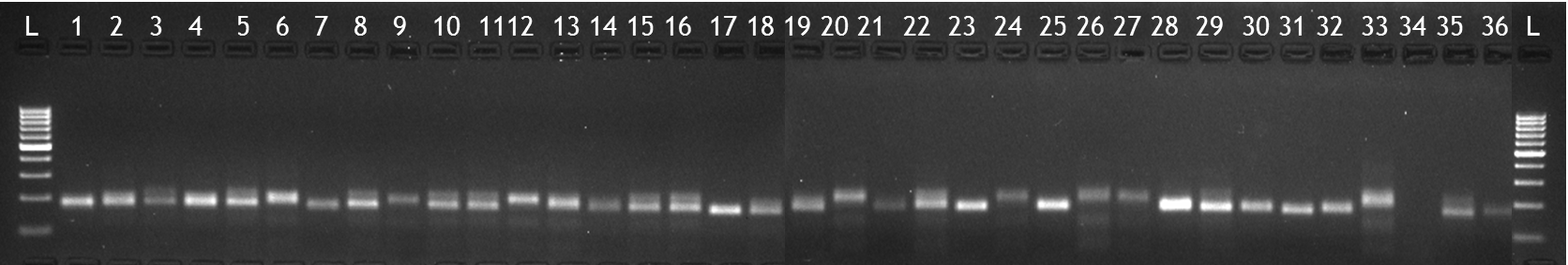

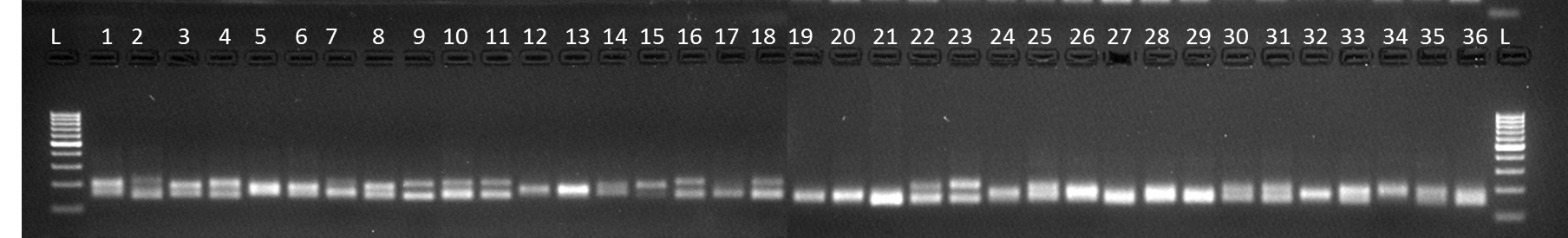


PCR amplification of 36 genotypes with primer number- TKM 1357-1358

PCR amplification of 36 genotypes with primer number- TKM 1226-1227

PCR amplification of 36 genotypes with primer number- TKM 1361-1362
