# Supplementary material for: TeaMiD: a comprehensive database of simple sequence repeat markers of tea
Source: Database (Oxford). 2020 Mar 11;2020:baaa013. doi: 10.1093/database/baaa013 (PMC7065459; doi:10.1093/database/baaa013)
Supplement: Supp_baaa013 [file supp_baaa013.zip › Supplimentray-Table-S12.doc]

Table S12. List of selected tea genotypes used in the present study.

| **S. no.** | **Accession** | **Morphotype** |
| --- | --- | --- |
| 1 | **CP1** | ChinaAssam |
| 2 | 19.55.49 | Assam hybrid |
| 3 | 19.60.10 | Assam hybrid |
| 4 | 19.58.17 | Assam hybrid |
| 5 | 19/56/27 | Assam hybrid |
| 6 | S.A. | Assam China |
| 7 | 19/56/42 | Assam hybrid |
| 8 | 19/57/36 | Assam hybrid |
| 9 | 19.55.46 | Assam hybrid |
| 10 | 651.63 | Assam hybrid |
| 11 | 651.25 | Assam hybrid |
| 12 | **P.133** | Assam |
| 13 | LV.18 | Assam |
| 14 | 19.61.25 | Assam hybrid |
| 15 | 651.32 | Assam hybrid |
| 16 | 651.36 | Assam hybrid |
| 17 | 19.60.39 | Assam hybrid |
| 18 | 651.57 | Assam hybrid |
| 19 | 19.60.45 | Assam hybrid |
| 20 | 19.61.4 | Assam hybrid |
| 21 | 19.61.33 | Assam hybrid |
| 22 | 651.71 | Assam hybrid |
| 23 | 19.59.20 | Assam hybrid |
| 24 | T.3E.3 | Assam hybrid |
| 25 | 19/56/21 | Assam hybrid |
| 26 | ***Camellia rosiflora*** | Camellia species |
| 27 | SS.28 | Assam China hybrid |
| 28 | Tingamira | Assam hybrid |
| 29 | Dhonjan | Assam hybrid |
| 30 | **128.32.8** | China type |
| 31 | **128.26.2** | China type |
| 32 | **270.3.11** | Cambod hybrid |
| 33 | Gourisankar | Assam hybrid |
| 34 | ***Camellia sasanqua*** | Wild Camellia species |
| 35 | **480.19** | Cambod |
| 36 | **Betjan** | Assam hybrid |

*The bold genotypes were taken for initial screening
