# Supplementary material for: TeaMiD: a comprehensive database of simple sequence repeat markers of tea
Source: Database (Oxford). 2020 Mar 11;2020:baaa013. doi: 10.1093/database/baaa013 (PMC7065459; doi:10.1093/database/baaa013)
Supplement: Supp_baaa013 [file supp_baaa013.zip › Supplimentray-Table-S13.doc]

| Primer number  Table S13: Randomly selected six polymorphic primer used in genetic diversity study of 36 Tea genotypes | Forward primer(5’-3’) | Reverse primer(5’-3’) | Annealing temperature (°C) | Number of alleles | PIC |
| --- | --- | --- | --- | --- | --- |
| TKM 1226-1227 | CACACCAAATTTTCCAGAACC | CCACGTTATATAACATGCAAGC | 58° | 5 | 0.718 |
| TKM 1357-1358 | TGTTATGCATCGAAGTTTGC | ACGTGTTGTTTCGGCATTGA | 58° | 5 | 0.750 |
| TKM 1361-1362 | CTCACAGTACAGAAATTGATGC | AAACTGTTTGAAGATGGAAGCC | 58° | 5 | 0.769 |
| TKM 1383-1384 | GTGGTGTCATAATTTATCC | ATTGTTGAACAGGTATGG | 54° | 6 | 0.613 |
| TKM 1421-1422 | GTGAACAATGGTAGTGTAAAGG | TCCTCTTGACTTTTTCTATGCC | 58° | 4 | 0.721 |
| TKM 1439-1440 | TCAATCACATGCAACCAGTGT | GCCCTCTTCCCAAACTCCTC | 56° | 5 | 0.718 |
