# Supplementary material for: TeaMiD: a comprehensive database of simple sequence repeat markers of tea
Source: Database (Oxford). 2020 Mar 11;2020:baaa013. doi: 10.1093/database/baaa013 (PMC7065459; doi:10.1093/database/baaa013)
Supplement: Supp_baaa013 [file supp_baaa013.zip › Supplimentray-Table-S5.doc]

Supplementary Table S5: SSR identified in important metabolic pathway genes.

| Pathway | No. of enzymes in pathway* | Enzyme name | No. of sequences annotated in CSS | No. of sequences annotated in CSA |
| --- | --- | --- | --- | --- |
| Flavone and flavonol biosynthesis | 2 | ec:3.2.1.31 - beta-glucuronide glucuronohydrolase glucuronidase | 1 | 0 |
|  |  | ec:2.4.1.91 - 3-O-glucosyltransferase | 1 | 0 |
| Flavonoid biosynthesis | 9 | ec:1.1.1.234 - 4-reductase | 1 | 0 |
|  |  | ec:1.17.1.3 - reductase | 2 | 1 |
|  |  | ec:2.1.1.104 - O-methyltransferase | 6 | 0 |
|  |  | ec:2.3.1.74 - synthase | 4 | 0 |
|  |  | ec:5.5.1.6 - isomerase | 4 | 2 |
|  |  | ec:1.3.1.77 - reductase [(2R 3R)-flavan-3-ol-forming] | 3 | 0 |
|  |  | ec:1.1.1.219 - 4-reductase | 6 | 0 |
|  |  | ec:2.3.1.133 - O-hydroxycinnamoyltransferase | 5 | 1 |
|  |  | ec:1.14.11.9 - 3-dioxygenase | 5 | 3 |
| Isoflavonoid biosynthesis | 2 | ec:1.3.1.45 - reductase | 2 | 0 |
|  |  | ec:4.2.1.105 - dehydratase | 1 | 1 |
| Phenylpropanoid biosynthesis | 17 | ec:3.2.1.126 - beta-glucosidase | 2 | 0 |
|  |  | ec:1.1.1.195 - dehydrogenase | 7 | 3 |
|  |  | ec:1.11.1.7 - lactoperoxidase | 81 | 19 |
|  |  | ec:1.2.1.68 - dehydrogenase | 2 | 0 |
|  |  | ec:4.3.1.24 - ammonia-lyase | 1 | 0 |
|  |  | ec:2.1.1.146 - O-methyltransferase | 0 | 1 |
|  |  | ec:2.1.1.104 - O-methyltransferase | 6 | 0 |
|  |  | ec:3.1.1.49 - esterase | 1 | 0 |
|  |  | ec:2.4.1.128 - glucosyltransferase | 4 | 1 |
|  |  | ec:2.3.1.92 - O-sinapoyltransferase | 1 | 0 |
|  |  | ec:1.2.1.44 - reductase | 14 | 0 |
|  |  | ec:2.3.1.99 - O-hydroxycinnamoyltransferase | 5 | 0 |
|  |  | ec:4.3.1.25 - ammonia-lyase | 1 | 0 |
|  |  | ec:3.2.1.21 - gentiobiase | 44 | 13 |
|  |  | ec:2.3.1.133 - O-hydroxycinnamoyltransferase | 5 | 1 |
|  |  | ec:2.1.1.68 - O-methyltransferase | 5 | 0 |
|  |  | ec:6.2.1.12 - ligase | 6 | 2 |
| Anthocyanin biosynthesis | 1 | ec:2.4.1.115 - 3-O-glucosyltransferase | 3 | 0 |
| Caffeine metabolism | 3 | ec:1.7.3.3 - urate hydroxylase | 2 | 1 |
|  |  | ec:2.1.1.160 - synthase | 3 | 0 |
|  |  | ec:2.3.1.5 - N-acetyltransferase | 61 | 0 |
| Indole alkaloid biosynthesis | 5 | ec:2.3.1.160 - synthase | 3 | 0 |
|  |  | ec:2.4.1.219 - glucosyltransferase | 1 | 0 |
|  |  | ec:4.3.3.2 - synthase | 5 | 0 |
|  |  | ec:2.1.1.99 - N-methyltransferase | 0 | 1 |
|  |  | ec:3.2.1.125 - beta-glucosidase | 1 | 0 |
| Isoquinoline alkaloid biosynthesis | 11 | ec:2.6.1.57 - transaminase | 2 | 5 |
|  |  | ec:2.6.1.5 - transaminase | 6 | 5 |
|  |  | ec:1.1.1.247 - reductase (NADPH) | 6 | 1 |
|  |  | ec:2.6.1.1 - transaminase | 3 | 6 |
|  |  | ec:1.4.3.2 - oxidase | 2 | 0 |
|  |  | ec:1.1.1.248 - reductase (NADPH) | 11 | 1 |
|  |  | ec:2.1.1.140 - (S)-coclaurine-N-methyltransferase | 3 | 1 |
|  |  | ec:1.10.3.1 - oxidase | 2 | 0 |
|  |  | ec:1.14.11.31 - 6-O-demethylase | 8 | 0 |
|  |  | ec:1.4.3.21 - oxidase | 6 | 0 |
|  |  | ec:1.14.11.32 - 3-O-demethylase | 10 | 0 |
| Tropane, piperidine and pyridine alkaloid biosynthesis | 10 | ec:1.1.1.237 - reductase | 3 | 1 |
|  |  | ec:2.6.1.57 - transaminase | 2 | 5 |
|  |  | ec:2.6.1.5 - transaminase | 6 | 5 |
|  |  | ec:2.6.1.1 - transaminase | 3 | 6 |
|  |  | ec:2.6.1.9 - transaminase | 2 | 5 |
|  |  | ec:2.6.1.58 - transaminase | 2 | 0 |
|  |  | ec:1.4.3.21 - oxidase | 6 | 0 |
|  |  | ec:1.1.1.206 - reductase I | 2 | 1 |
|  |  | ec:1.1.1.236 - reductase II | 1 | 1 |
|  |  | ec:2.3.1.93 - O-tigloyltransferase | 1 | 0 |
| Biosynthesis of secondary metabolites - unclassified | 1 | ec:2.3.1.30 - O-acetyltransferase | 2 | 0 |

*Total number of enzymes identified from CSS and CSA genomes for the related pathway.
